# Supplementary material for: Physical therapy in the intensive care unit: A cross-sectional study of three Asian countries
Source: PLoS One. 2023 Nov 9;18(11):e0289876. doi: 10.1371/journal.pone.0289876 (PMC10635439; doi:10.1371/journal.pone.0289876)
Supplement: S1 Table — (PDF) [file pone.0289876.s001.pdf]

S1 Table. Most commonly implemented PT techniques in the ICU

n (%)	**JP** (n=76)	**PH** (n=45)	**TW** (n=43)	***p***
Breathing exercise	6 (7.89)	35 (77.78)	26 (60.47)	**0.000**[a]
Chest expansion technique	7 (9.21)	11 (24.44)	24 (55.81)	**0.000**[a]
Incentive Spirometry	0 (0)	7 (15.56)	6 (13.95)	**0.003**[a]
Forced expiration technique	3 (3.95)	4 (8.89)	3 (6.98)	0.541[b]
Active cycle of breathing	3 (3.95)	4 (8.89)	2 (4.65)	0.564[b]
Autogenic drainage	2 (2.63)	2 (4.44)	0 (0)	0.380[b]
Postural drainage	53 (69.74)	7 (15.56)	6 (13.95)	**0.000**[a]
Suction technique	7 (9.21)	1 (2.22)	3 (6.98)	0.350[b]
Percussion technique	0 (0)	6 (13.33)	8 (18.6)	**0.000**[b]
Vibration technique	1 (1.32)	7 (15.56)	6 (13.95)	**0.003**[b]
Coughing technique	8 (10.53)	8 (17.78)	14 (32.56)	**0.012**[b]
Huffing technique	1 (1.32)	4 (8.89)	5 (11.63)	0.051[b]
Positioning	34 (44.74)	39 (86.67)	30 (69.77)	**0.000**[a]
Passive ROM	44 (57.89)	42 (93.33)	36 (83.72)	**0.000**[a]
Active-Assisted ROM	25 (32.89)	36 (80)	35 (81.4)	**0.000**[a]
Active ROM	9 (11.84)	31 (68.89)	26 (60.47)	**0.000**[a]
Stretching exercise	11 (14.47)	21 (46.67)	14 (32.56)	**0.000**[a]
Progressive resistance exercise	20 (26.32)	12 (26.67)	2 (4.65)	**0.010**[a]
Electrotherapy	9 (11.84)	32 (71.11)	2 (4.65)	**0.000**[a]
Bicycle ergometer	2 (2.63)	8 (17.78)	3 (6.98)	**0.011**[b]
Sitting balance & tolerance	72 (94.74)	26 (57.78)	16 (37.21)	**0.000**[a]
Standing balance & tolerance	48 (63.16)	13 (28.89)	8 (18.6)	**0.000**[a]
Ambulation training	30 (39.47)	8 (17.78)	2 (4.65)	**0.000**[a]
Others	3 (3.95)	1 (2.22)	4 (9.3)	0.267[b]

n, number; %, percentage; *, frequency based on those who answered ‘yes’; #, multiple answer; JP, Japan; PH, Philippines; TW, Taiwan; ICU, Intensive Care Unit; PT, Physical Therapy; bolded numbers signify $p<0.05$

[a] Pearson Chi-Square Test

[b] Fisher’s Exact Test
