# Supplementary material for: Physical therapy in the intensive care unit: A cross-sectional study of three Asian countries
Source: PLoS One. 2023 Nov 9;18(11):e0289876. doi: 10.1371/journal.pone.0289876 (PMC10635439; doi:10.1371/journal.pone.0289876)
Supplement: S2 Table — (PDF) [file pone.0289876.s002.pdf]

S2 Table. Challenges in the delivery of PT services in the ICU

n (%)	**JP** (n=76)	**PH** (n=45)	**TW** (n=43)	***p***	p^c
No direct access to ICU patients	39 (51.32)	9 (20)	8 (18.6)	**0.000**[a]	0.0502
PT-ICU patient ratio	25 (32.89)	8 (17.78)	8 (18.6)	0.095[a]	0.2272
Little-to-no training prior to ICU duty	44 (57.89)	21 (46.67)	21 (48.84)	0.333[a]	**0.0084**
Decreased PT ICU exposure	3 (3.95)	16 (35.56)	14 (32.56)	**0.000**[a]	**0.0103**
Little-to-no autonomy in treatment modification	5 (6.58)	10 (22.22)	6 (13.95)	**0.044**[a]	0.0854
Limited PT knowledge allowed to conduct to ICU patients	12 (15.79)	9 (20)	13 (30.23)	0.173[a]	0.1510
Provision of PT by ICU nurses instead of PTs perse	6 (7.89)	8 (17.78)	1 (2.33)	**0.043**[b]	0.8885
Delayed PT service provision	6 (7.89)	8 (17.78)	8 (18.6)	0.155[a]	0.1127

n, number; %, percentage; #, multiple answer; JP, Japan; PH, Philippines; TW, Taiwan; ICU, Intensive Care Unit; PT, Physical Therapy; PTs, physical therapist; bolded numbers signify $p<0.05$

[a] Pearson Chi-Square Test

[b] Fisher's Exact Test

[c] Using logistic regression with adjustment of age, highest educational attainment, and work experience as physiotherapist
