# Supplementary material for: Physical therapy in the intensive care unit: A cross-sectional study of three Asian countries
Source: PLoS One. 2023 Nov 9;18(11):e0289876. doi: 10.1371/journal.pone.0289876 (PMC10635439; doi:10.1371/journal.pone.0289876)
Supplement: S1 Checklist — (DOCX) [file pone.0289876.s003.docx]

**STROBE Statement – Checklist of items that should be included in reports of cross-sectional studies.**

|  | **Item No.** | **Recommendation** | **Yes/No** | **Page No.** |
| --- | --- | --- | --- | --- |
| **Title and abstract** | 1 | (a) Indicate the study’s design with a commonly used term in the title or the abstract | Yes | 1-2 |
|  |  | (b) Provide in the abstract an informative and balanced summary of what was done and what was found | Yes | 1-2 |
| **Introduction** |  |  |  |  |
| Background rationale | 2 | Explain the scientific background and rationale for the investigation being reported. | Yes | 3-5 |
| Objectives | 3 | State specific objectives, including any prespecified hypotheses | Yes | 5 |
| **Methods** |  |  |  |  |
|  |  |  |  |  |
| Study design | 4 | Present key elements of study design early in the paper | Yes | 5 |
| Setting | 5 | Describe the setting, location, and relevant dates, including periods of recruitment, exposure, follow-up, and data collection | Yes | 6 |
| Participants | 6 | Give the eligibility criteria, and the sources and methods of selection of participants | Yes | 6 |
| Variables | 7 | Clearly define all outcomes, exposures, predictors, potential confounders, and effect modifiers. Give diagnostic criteria, if applicable |  |  |
| Data sources/ measurement | 8* | For each variable of interest, give sources of data and details of methods of assessment (measurement). Describe comparability of assessment methods if there is more than one group | Yes | 6-7 |
| Bias | 9 | Describe any efforts to address potential sources of bias |  |  |
| Study size | 10 | Explain how the study size was arrived at | Yes | 6-7 |
| Quantitative variables | 11 | Explain how quantitative variables were handled in the analyses. If applicable, describe which groupings were chosen and why | Yes | 6-7 |
| Statistical methods | 12 | (a) Describe all statistical methods, including those used to control confounding | Yes | 6-7 |
|  |  | (b) Describe any methods used to examine subgroups and interactions | Yes | 6-7 |
|  |  | (c) Explain how missing data were addressed | No | - |
|  |  | (d) If applicable, describe analytical methods taking account of sampling strategy | Yes | 6-7 |
|  |  | (e) Describe any sensitivity analyses | No | - |
| **Results** |  |  |  |  |
| Participants | 13* | (a) Report numbers of individuals at each stage of the study – eg numbers potentially eligible, examined for eligibility, confirmed eligible, included in the study, completing follow-up, and analyzed | Yes | 7  (Fig 1) |
|  |  | (b) Give reasons for non-participation at each stage | Yes | 7  (Fig 1) |
|  |  | (c) Consider use of a flow diagram | Yes | 7 |
| Descriptive data | 14* | (a) Give characteristics of study participants (eg, demographic, clinical, social) and information on exposures and potential confounders | Yes | 7-9 |
|  |  | (b) Indicate number of participants with missing data for each variable of interest | Yes | 7-14 |
| Outcome data | 15* | Report numbers of outcome events or summary measures | Yes | 7-14,  S1 Table, S2 Table |
| Main results | 16 | 1. Give unadjusted estimates and, if applicable, confounder-adjusted estimates and their precision (eg, 95% confidence interval). Make clear which confounders were adjusted for and why they were included. | Yes | 7-14, S  1 Table, S2 Table |
|  |  | 1. Report category boundaries when continuous variables were categorized. | NA | NA |
|  |  | (c) If relevant, consider translating estimates of relative risk into absolute risk for a meaningful time period. | NA | NA |
| Other analyses | 17 | Report other analyses done – e.g. analyses of subgroups and interactions, and sensitivity analyses | NA | NA |
| **Discussion** |  |  |  |  |
| Key results | 18 | Summarise key results with reference to study objectives | Yes | 17 |
| Limitations | 19 | Discuss limitations of the study, taking into account sources of potential bias or imprecision. Discuss both direction and magnitude of any potential bias | Yes | 25-26 |
| Interpretation | 20 | Give a cautious overall interpretation of results considering objectives, limitations, multiplicity of analyses, results from similar studies, and other relevant evidence. | Yes | 17-26 |
| Generalisability | 21 | Discuss the generalizability of the study results | Yes | 26 |
| **Other Information** |  |  |  |  |
| Funding | 22 | Give the source of funding and the role of the funders for the present study and, if applicable, for the original study on which the present article is based. |  | 26-27 |

NA, not applicable
