# Supplementary material for: Physical therapy in the intensive care unit: A cross-sectional study of three Asian countries
Source: PLoS One. 2023 Nov 9;18(11):e0289876. doi: 10.1371/journal.pone.0289876 (PMC10635439; doi:10.1371/journal.pone.0289876)
Supplement: Attachment — Submitted filename: Response to Reviewers.docx [file pone.0289876.s007.docx]

Reviewers' comments:

Reviewer #1: Dear Authors
Thanks a lot for the opportunity you have offered me to revise again the fascinating manuscript " Physical therapy in the intensive care unit: A comparative study of three Asian countries". I thank the authors for having improved the manuscript following the suggestions of reviewers.
Despite the ameliorations, I invite the authors to add the reference of the STROBE and CHERRIES in the main manuscript.
Thus, after having resolved these minor revisions, the paper will be ready to be accepted and published.
I am available to revise the paper again.
Best regards.

Response: Thanks for reviewer’s suggestion. We have added the references of STROBE and CHERRIES in manuscript.

Revised:

Line 109:

We used a two-part, semistructured, open online survey which based on Equator Network's Checklist for Reporting Results of Internet E-Surveys (CHERRIES) guidelines [29].

Line 125:

The final survey was evaluated with the Strengthening the Reporting of Observational Studies in Epidemiology (STROBE) statement [30] (S3).

Reviewer #3: Dear author, here are my suggestions:
1. I suggest replacing the term “observational comparative” with “cross-sectional study” in all the manuscript

Response: Thanks for reviewer’s suggestion. We have changed all “observational comparative” with “cross-sectional study” in manuscript.

2. Please, insert in the method section all the reporting guidelines that you have used to write the manuscript (e.g. “This protocol is reported in accordance with …)

Response: Thanks for reviewer’s suggestion. We have added the references of CHERRIES in manuscript.

Revised:

Line 109

We used a two-part, semistructured, open online survey which based on Equator Network's Checklist for Reporting Results of Internet E-Surveys (CHERRIES) guidelines [29].

3. In the method section, please justify how you decided to use Fisher’s exact test instead of the chi–square test. Also, specify how you evaluated all the statistical test assumptions.

Response: Thanks for reviewer’s suggestion. We have added the description of when to use Fisher’s exact test at “Statistical Analyses” section.

Revised:

Line 127

The data were analyzed using IBM SPSS Statistics for Windows, Version 22.0. (Armonk, NY: IBM Corp) and SAS 9.4 (SAS Institute Inc., Cary, NC, USA). The respondents’ sociodemographic and ICU-related characteristics are presented as frequency (f), percentage (%), and mean. For the categorical variables, chi-square or Fisher’s exact test were used, and Fisher’s exact test was conducted instead of chi-square test if any of the expected values in a cell is less than five. The one-way analysis of variance (ANOVA) was used for continuous variables. The level of significance was set at p < 0.05. Although the sample size was not determined, sample size between 30 and 500 for comparative analysis was considered appropriate [31].

4. The study suffers from a possible selection bias. Indeed, physical therapists (PT) who use social media do not represent all the PT who work in an ICU setting (e.g., probably younger and more prone to use online resources for their training). Therefore, I suggest inserting this limitation of the study in the discussion.

Response: Thanks for reviewer’s suggestion. We have added this limitation at “Discussion” section.

Revised:

Line 416

Fourth, there were potential selection bias due to links of surveys in Taiwan and Philippines were post on social media since therapists who use social media may be relatively more proactive in answering online questionnaires or younger.

5. The statistical analysis conducted (univariable hypothesis testing) does not permit to conclude that the interventions applied in the ICU setting are different because of country differences. The sample of PT in the three countries analyzed is different for significant variables such as age and PT-patient ratio. Therefore, there are many confounders in the relationship of PT intervention – country. I suggest conducting a regression analysis adjusting for possible cofounders to determine the predictors of the different interventions applied. Otherwise (e.g., the sample size is too small to perform a regression), I suggest inserting the lack of an adjustment in the analysis as a major limitation of the study.

Response: Thanks for reviewer’s suggestion. We have assessed data of “challenges” using logistic regression with adjustment of “age”, “highest educational attainment “, and “work experience as physiotherapist”. Indeed, after adjustment, there are several data became non-significant different after logistic regression. Thus, we added one paragraph in result section.

Revised:

Line 225:

Due to different sociodemographic profile between three countries, we further assessed whether there was difference in each challenges using logistic regression with adjustment of “age”, “highest educational attainment “, and “work experience as physiotherapist”. After adjustment, there is no significantly difference between three countries in “No direct access to ICU”, “Little-to-no autonomy in treatment modification”, and “Provision of PT by ICU nurses instead of PTs perse patients”. However, the p value of “No direct access to ICU” is near 0.05 (p = 0.0502). Moreover, “Little-to-no training prior to ICU duty” was changed from non-significantly to significantly, and “Decreased PT ICU exposure” remains still significantly different between three countries. In “Little-to-no training prior to ICU duty”, the odd ratio of Philippines vs Japan and Taiwan vs Japan are 0.154 and 0.376, respectively. These indicate Japanese therapists considered lack of training as a bigger challenge than therapists in the other two countries.
