# Supplementary material for: Physical therapy in the intensive care unit: A cross-sectional study of three Asian countries
Source: PLoS One. 2023 Nov 9;18(11):e0289876. doi: 10.1371/journal.pone.0289876 (PMC10635439; doi:10.1371/journal.pone.0289876)
Supplement: Attachment — Submitted filename: Reviewer Comments .docx [file pone.0289876.s006.docx]

Reviewer #1:

Major revisions

1. I suggest that authors adhere to the suggested reporting for STROBE observational studies. (https://www.thelancet.com/journals/lancet/article/PIIS0140-6736(07)61602-X/fulltext#article_upsell).

Response: Thanks for reviewer’s suggestion. We have added STROBE Statement – Checklist as a supplementary document.

2. If their survey is electronic, I recommend that they also use the reporting of CHERRIES (https://www.ncbi.nlm.nih.gov/pmc/articles/PMC1550605/).

Response: Thanks for reviewer’s suggestion. We have used reporting of CHERRIES to check our manuscript. However, not all items were mentioned in manuscript.

3. Authors should reorganize the manuscript following these guidelines for the sections materials and methods, results and discussion.

Response: Thanks for reviewer’s suggestion. We have re-reorganize the manuscript following guidelines.

3.1 They should declare clearly the adoption of this guideline in the Methods section.

Response: Thanks for reviewer’s suggestion. The survey was based on the study of Sigera et al. on 2016. We removed questions of ICU research and on-call service. In addition, we modified/included several questions such as the type of hospital, work experience, most common physiotherapy interventions, and common challenge experienced in the ICU.

3.2 How many questions made up each section?

Response: Thanks for reviewer’s suggestion. There are two sections and total 21 questions.

- Section I, 7.
- Section II, 14.

3.3. What type of questions were used?

Response: Type of questions used identified. *(line 119)*

3.4. How many answer possibilities were there?

Response: The answer possibilities are different in each question. All are multiple answeres.

3.5. How many pages were there?

Response: There were a total of eight (8) sections in the google form including the informed consent section and instruction section.

3.6. Survey testing. Was the survey tested with a pilot? Please be specific.

Response: No pilot-testing of the survey made and hence included as a limitation.. However, the e-survey of Taiwanese version was validated by two PTs who currently work in ICU. After they gave suggestion, some questions were edited.

3.6. Administration. How was the survey administered (paper or online)? How were the participants contacted? Was it free to fill out, or was there an obligation? How long did it last? Were there any economic incentives for completion? How was the possibility of multiple compilations avoided?

Response: The survey is e-survey. Responses were collected via Google Forms. The link of electronic surveys was posted on social media of PT interest groups in Taiwan and Philippine, and Japanese Society of Education for Physicians and Trainees in Intensive Care (JSEPTIC) in Japan. The Google Forms were opened from November 2021 to February 2022. There was no economic incentives and the e-survey is free to fill out. Once the answer was sent, there was no possibility for modification.

3.7. Analysis of the survey. How were missing data handled?

Response: All participants completed the survey. No missing data was needed for handling.

Minor revisions
4. TITLE: I suggest authors add in the title the study design (e.g., : an observational study)

Response: Thanks for reviewer’s suggestion. We have changed title to “Physical therapy in the intensive care unit: An observational comparative study of three Asian countries”

5. ABSTRACT: result. Please add statistical values to sustain your statement (e.g., %, n= …).
Response: Thanks for reviewer’s suggestion. Abstract was revised.

5. KEYWORDS: I suggest authors add "rehabilitation", "physical medicine" "Physiotherapists", "survey".
Response: Keywords were added

6. RESULTS: The rate of response is low. This point is a limitation of authors' study. I suggest they declare it in the limitation section.

Response: Low response rate is included in the limitation.

7. DISCUSSION: General. The discussion is well organized, but it is a little bit longer. I suggest authors shorten and focus more on it. For example, it is better to avoid the repetition of the results. Discussing the findings with existing evidence or comparing them with different scenarios (e.g., European and American countries) is suitable.

Response:

- As the STROBE checklist was suggested to be used, a summary of the study was included in the first part of the discussion. Nevertheless, the longer version of the summarized part was cut short to only two sentences.
- Sociodemographic profile was cut a little shorter.
- The repetition in discussion was removed.

8. Direct access of PT. The topic of direct access in physiotherapy is very relevant and strongly discussed also in other fields (e.g. musculoskeletal, e.g. Maselli et al. Direct Access to Physical Therapy: Should Italy Move Forward? Int J Environ Res Public Health. 2022 Jan 4;19(1):555. doi: 10.3390/ijerph19010555). I think this point needs to be developed by emphasising that there is a call to action at international level to develop direct access patient management skills.

Response:

- Due to the medical regulations in JP, TW, and PH, there is no direct access except certain conditions such as health promotion and sports injury prevention.
- Suggestion to emphasize that a call to action regarding direct access was included. (line 380)

9. Interprofessional work. Authors have reported the importance of interprofessional work (e.g., between physiotherapists and nurses) in the ICU setting. This element emerged strongly also from different qualitative studies involving nurses, especially during the COVID-19 pandemic. For example, Fontanini et al. have reported the importance of interprofessional work in the ICU setting. I suggest authors look at this paper for their discussion (Italian Nurses' experiences during the COVID-19 pandemic: a qualitative analysis of internet posts. Int Nurs Rev. 2021 Jun;68(2):238-247. doi: 10.1111/inr.12669).

Response: Thanks for reviewer’s suggestion. We have added the importance of interprofessional work in the ICU.

10. Managerial perspectives. Authors frequently suggest practical actions for managers. Some of these actions are similar to those proposed for emotional intelligence leaders in managing complex healthcare scenarios (e.g., improve communication, awareness, multidisciplinary works, etc). I suggest authors look at this recent publication in the rehabilitation field by Dell'Isola's research group. Maybe, this publication can add some points to the authors' discussion. (COVID-19 and Health Care Leaders: How Could Emotional Intelligence Be a Helpful Resource During a Pandemic? Phys Ther. 2021 Sep 1;101(9):pzab143. doi: 10.1093/ptj/pzab143).

Response: Thanks for reviewer’s suggestion. Included how good relationship among members of the HC team could help improve work and patient-related outcome. (line 390)

11. Limitations. The rate of response is low. This point is a limitation of the authors' study. I suggest they declare it in the limitation section. Moreover, in this section, the authors should call for future research in the field aimed at overcoming the limits presented in their study.

Response: We have Included the “low response rate” as limitation.

12. CONCLUSION: Implications. According to their findings, I suggest authors organize this section in implications for clinical practice, education and management. This will be very informative.

Response: Thanks for reviewer’s suggestion. Included clinical implications. *(lines 413-416)*

13. LANGUAGE: There is a need for a language revision by a native English speaker. For example, there are different grammar mistakes. Moreover, various too many long sentences are presented, limiting the manuscript's readability. Please have a look at the language of the manuscript.

Response: The revised manuscript has been edited by professional English editors.

Reviewer #2:

Dear Author,
1. First of all, why did the author choose few Asian countries and the major flaw is validity of the questionnaire used. The questionnaire was not translated as per the standard protocol.

Response: Thanks for reviewer’s suggestion. We chose these three Asia countries due to Japan, Philippines, and Taiwan have different healthcare system, and there is no study regarding the current status of PT practice in the ICU. About translation, yes, questionnaires was no translated according to standard protocol. However, Professor Takahashi, also as a PT, is the Executive Director of The Japanese Society of Intensive Care Medicine. We believe his participation is sufficient to increase the validity of the questionnaire.
2. The sample size is small.

Response: Thanks for reviewer’s suggestion. Included small sample size seen as low response rate which is included in the limitation.

3. The justification to perform the study is not provided

Response: Although numerous studies have reported on the current status of PT intervention in the ICU, no study has compared it between different Asian countries. Since Japan, Philippines, and Taiwan have different health-care systems, we compared the extent of PT intervention in the ICU between these three countries.
